# Antiviral interferons induced by Newcastle disease virus (NDV) drive a tumor-selective apoptosis

**DOI:** 10.1038/s41598-019-51465-6

**Published:** 2019-10-22

**Authors:** Teridah Ernala Ginting, Salomo Christian, Young Othiwi Larasati, Jeremiah Suryatenggara, Ivet Marita Suriapranata, George Mathew

**Affiliations:** Division of Immunology, Mochtar Riady Institute for Nanotechnology and Medical Science Group, University of Pelita Harapan, Jalan Boulevard Jenderal Sudirman 1688, Lippo Karawaci, Tangerang, Banten 15811 Indonesia

**Keywords:** Tumour virus infections, Cytokines, Tumour immunology, Viral host response

## Abstract

Newcastle disease virus (NDV) strongly induces both type I and III antiviral interferons (IFNs-α/-β and IFN-λ, respectively) in tumor cells while it induces mainly type III IFN in normal cells. Impairment of antiviral type I IFN signaling in tumor cells is thought to be the reason for effective oncolysis. However, there is lack of clarity why lentogenic strain NDV can also induce oncolysis. NDV infection caused apoptosis in normal and tumor cells as demonstrated with the caspase-3 enzyme activation and annexin-V detection. The apoptosis response was inhibited by B18R protein (a type I IFN inhibitor) in tumor cells i.e. A549 and U87MG, and not in normal cells i.e. NB1RGB and HEK293. Similarly, UV-inactivated medium from NDV infection was shown to induce apoptosis in corresponding cells and the response was inhibited in A549 and U87MG cells with the addition of B18R protein. Treatment with combination of IFNs-α/-β/-λ or IFNs-α/-β or IFN-λ in NB1RGB, HEK293, A549 and U87MG showed that caspase activity in IFNs-α/-β/-λ group was the highest, followed with IFN-α/-β group and IFN-λ group. This suggests that tumor-selectivity of NDV is mainly because of the cumulative effect of type I and III in tumor cells that lead to higher apoptotic effect.

## Introduction

Potential use of oncolytic viruses are being evaluated as anticancer agent due to their selective killing of tumor cells with the minimal damage to normal cells. Among the studied oncolytic viruses, Newcastle disease virus (NDV) is a bird pathogen from the family Paramyxoviridae, and the only kind which is naturally does not infect mammalian. Due to its safety, efficacy and tumor-selective properties, NDV has been investigated as a novel oncolytic agent *in vitro*, *in vivo* as well as in clinical trials^1–10^

Virus infection results in signals to release antiviral interferons (IFN)^11–13^. These signals can be recognized by the membrane bound Toll-like receptors (TLR) that are involved in sensing viral glycoprotein, and the cytoplasmic RIG-like receptors (RLR) that is mainly responsible for sensing RNA viruses. This process activates transcription factors, most often interferon regulatory factor (IRF)3, IRF7 and nuclear factor kappa B (NFκB), that subsequently initiates transcription and expression of IFNs type I and III^12,14,15^. IFNs trigger many signaling pathways that modulate biological responses including antiviral, pro-apoptosis, tumor-suppressive and immunomodulatory effects in autocrine and paracrine fashion^16–23^. There are 3 major types of IFN: type I IFNs consists of 17 members including IFN-α, -β, ω, ε and κ that signal through IFNAR (interferon-alpha/beta receptor); type II IFN consists of IFN-γ that signals through IFNGR (interferon-gamma receptor); and type III IFNs consists of IFN-λ1, -λ2, -λ3 and -λ4 that signal through IFNLR1 (interferon-lambda receptor 1). Type I and III IFNs show similar antiviral effect through similar signaling pathway. Upon binding to their corresponding receptors, both type of IFNs will activate the Janus Kinase (JAK) and Signal Transduction and Activation of Transcription (STAT) proteins followed by transcription of ISGs (interferon stimulated genes)^24^.

NDV is a strong inducer of antiviral type I and III IFNs in human cell lines, with stronger induction of IFN-α and -β was observed in tumor cells relative to normal cells^25^. Different interferon signaling pathway between normal and tumor cells was the initiation of NDV tumor-selectivity mechanism. Unlike the normal cells, tumor cells exhibit impaired antiviral IFN signaling, in particular to type I IFNs, which allows virus to replicate efficiently. The defects in tumor cells can be deregulated IFN receptor expression, non-responsiveness to antiviral enzymes and impairment of STAT1 signaling^26–29^. Despite its inability to replicate, lentogenic non-lytic NDV strain at low MOI still possesses oncolytic ability; and many tumor cells which do not display defective IFN yet still are killed by NDV^25,30–32^.

Apoptosis is a multi-pathway program that leads to cell death. It can generally be divided into two nonexclusive signaling cascades involving death receptors (extrinsic pathways) or mitochondria (intrinsic pathway). In both pathways, cysteine aspartyl-specific proteases (caspases) that cleave cellular substrates are activated. NDV induces apoptosis through both pathways, although primarily through mitochondrial pathway mediated by induction of endoplasmic reticulum stress, release of cytochrome c and increase expression of CD95^22,33–37^. It has also been shown that NDV induced higher apoptosis in cells overexpressing antiapoptotic Bcl protein and in melanoma cells overexpressing antiapoptotic Livin protein^30,38^. IFN-α induces apoptosis in malignant cells through the activation of caspases-1, 2, 3, 8 and 9 and independent of Fas signaling^39^. Furthermore, IFN-α and -β induce apoptosis through the upregulation of transcriptional activity of p53 tumor suppressor. Consequently, little is known for the preferential lysis of the tumor cells in regards of distinct IFNs secretion induced by NDV.

In our previous study we observed the cytotoxicity and tumor selectivity of NDV and a difference in antiviral type I and III secretion in both normal and tumor cells following NDV infection^25^. In this study we examine the role of distinct IFNs in inducing apoptosis. We hypothesize that there is a tumor selective mechanism with regard of NDV-induced IFN and this mechanism further triggers apoptosis in tumor cells.

## Results

### NDV at 0.001 MOI did not replicate in tumor and normal cells

**Figure 1:**
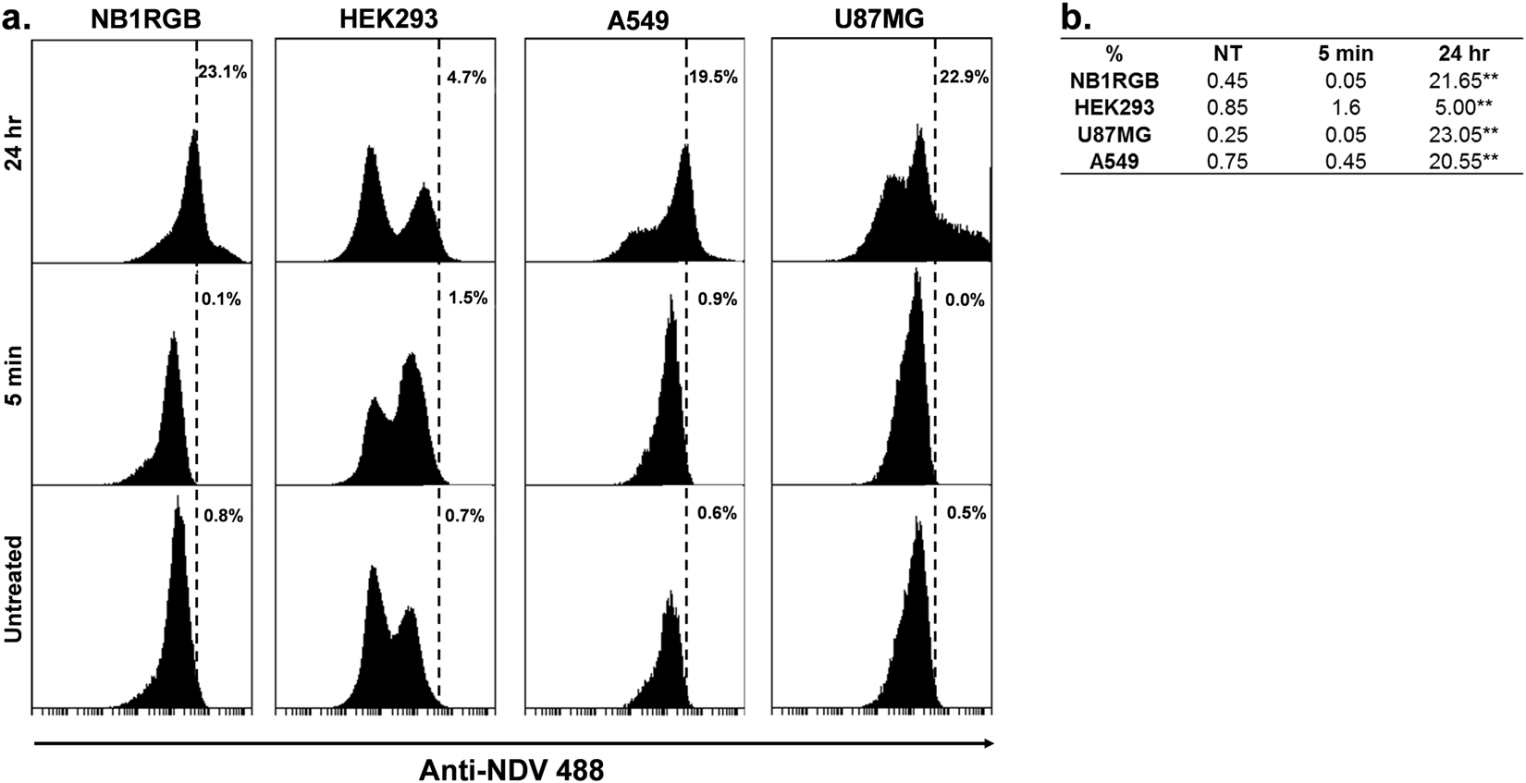
NDV protein expression on normal and tumor cells surface. (**a**) Flow cytometry analysis image of each cell types was representative from two or three replicates. Normal (NB1RGB and HEK293) and tumor (A549 and U87MG) cell lines were infected with 0.001 MOI NDV. NDV antigen was evaluated at 5 minutes (immediately) or 24 hours pi (5 min and 24 hr, respectively). Untreated group was noninfected wells and the cells were collected together with 5 min group for flow cytometry analysis. The statistical analysis of all data was presented in (**b**). Average (%) is percentage NDV positive cells from at least duplicate wells. *P ≤ 0.05, **P < 0.01.

In order to demonstrate that antitumor effect of NDV was caused by induced proinflammatory response rather than direct effect of virus replication, we used lentogenic NDV at 0.001 MOI for infection. We used this dose because it was enough to induce high IFNs production in our previous study^25^. We infected NB1RGB, HEK293, A549 and U87MG cells at for 72 hours. Supernatant from each cell was collected every 24 hours and titrated for virus TCID_50_ according to the relative fluorescence level. Virus titer in the medium samples of all cell type tested, were less than 1 log_10_ TCID_50_ from each time point (24, 48 and 72 hours p.i.) (Table 1). Flow cytometry analysis and immunofluorescence staining of NDV antigen on cell surface showed that all infected cells expressed NDV protein at 24 hours p.i. (Figs 1a,b, S1). However, because TCID_50_ result did not show positive wells, we confirmed that virus was not released to the medium at 0.001 MOI.

**Table 1:** Replication of NDV at 0.001 MOI in normal and tumor cells.

Cell name		Tissue	Log_10_ TCID_50_ hours p.i.		
			24	48	72
Normal	NB1RGB	Skin fibroblast	<1	<1	<1
	HEK293	Transformed embryonic kidney	<1	<1	<1
Tumor	A549	Non-small lung carcinoma	<1	<1	<1
	U87MG	Glioblastoma multiforme	<1	<1	<1

Virus titer at each time point was measured with fifty-percent endpoint method according to Reed and Muench^48^.

### NDV causes apoptosis in tumor cells and it is inhibited by B18R, a type I IFN inhibitor

**Figure 2:**
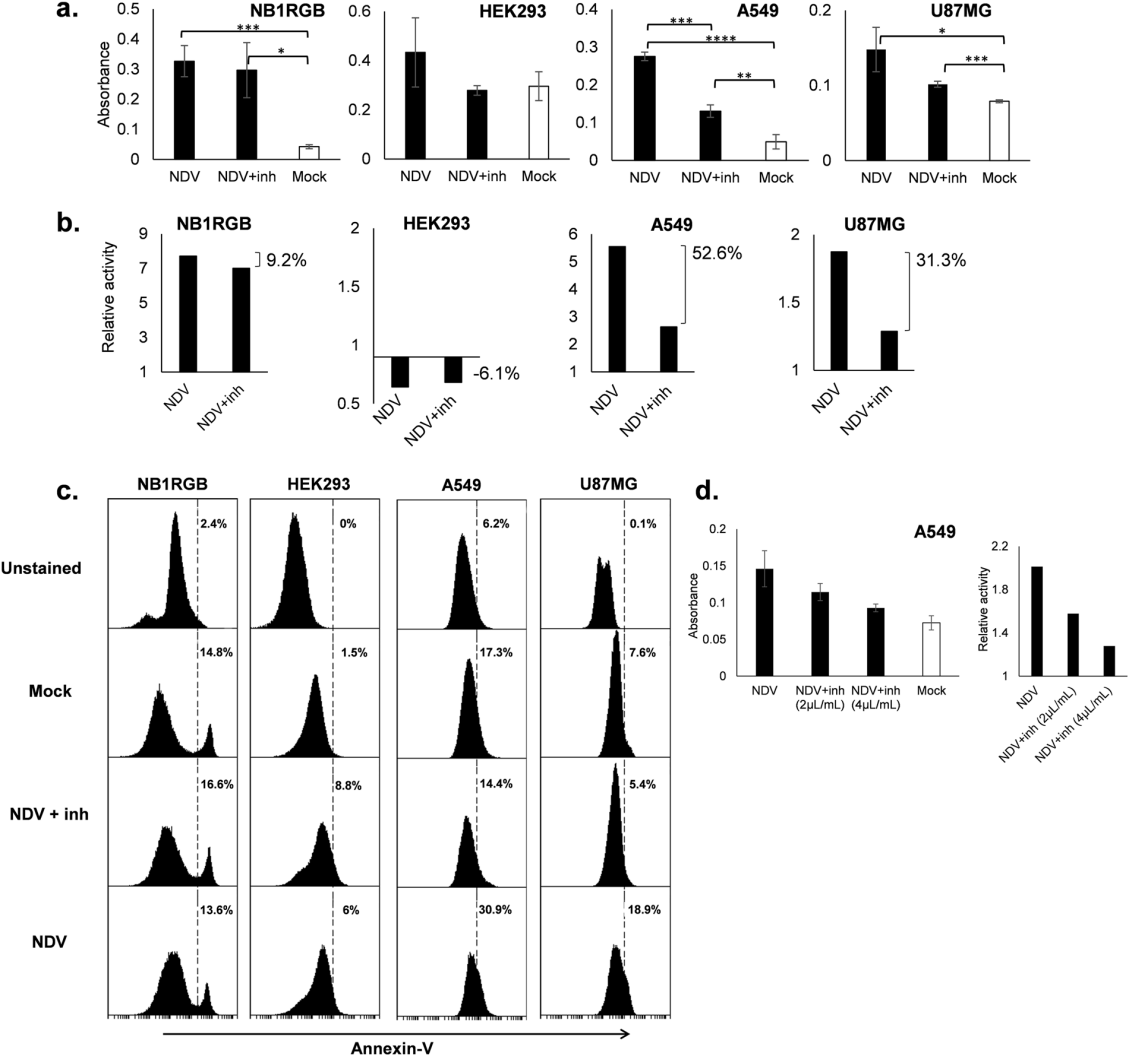
Apoptosis in normal and tumor cells after NDV infection. Apoptosis activity in normal cells, NB1RGB and HEK293, and tumor cells, U87MG and A549, after infection with NDV or NDV-B18R complex (NDV + inh) for 24 hours or 36 hours (for U87MG) (**a**). (**b**) is the relative of treatment to mock group from data (**a**) and percentage of inhibition is noted. (**c**) is annexin V flow cytometry analysis of normal cells, NB1RGB and HEK293, and tumor cells, U87MG and A549. (**d**) is dose-dependent apoptosis inhibition by B18R protein in A549 cells. Error bar is standard deviation from triplicate wells. *P ≤ 0.05, **P < 0.01, ***P < 0.001, ****P < 0.0001.

We previously observed that NDV was able to cause higher cytotoxicity in tumor cell lines rather than normal cell lines, by using MTT (3-(4,5-dimethylthiazol-2-Yl)-2,5-diphenyltetrazolium bromide) reagent^25^. In continuation with our former study, here we investigated if the cytotoxicity post NDV infection was an apoptosis process. Colorimetric assay was used to measure the cleaved caspase-3 enzyme. The results were presented in absorbance graph (Fig. 2a) to show significance level; and relative activity graph (2B) to omit different absorbance intensity in each group. We infected normal (NB1RGB and HEK293) and tumor cells (A549 and U87MG) with 0.001 MOI NDV to observe apoptosis activity. We used 0.001 MOI NDV at which the dose had been shown insufficient for NDV to replicate in both normal and tumor cells (Table 1) but was enough to induce high proinflammatory cytokines^25^, and caspase activity higher than apoptosis inducer such as staurosporine (Fig. S2).

Analysis of cell groups from NDV infection revealed significant caspase-3 activity in NB1RGB, A549 and U87MG cell lines, although absorbance activity in U87MG cells was lower. This suggested that NDV induced apoptosis on those cells tested, and different response between cell types might be present. On the other hand, NDV infection in HEK293 cells did not show any significant difference on caspase-3 activity (Fig. 2b,c).

To determine whether type I IFN was responsible for the observed apoptosis activity, we included treatment of B18R protein to same cells. B18R protein is a type I IFN inhibitor expressed by vaccinia virus^40,41^. Higher caspase-3 activity inhibition by B18R supplementation was seen in A549 and U87MG tumor cells (52.6% and 31.3%, respectively) rather than in NB1RGB and HEK293 normal cells (9.2% and −6.1%, respectively) as seen in Fig. 2b, although the significant difference in Caspase-3 activity between NDV and NDV-inhibitor groups was only observed in A549 cells (Fig. 2a).

This result suggested a type I IFN role in apoptosis in A549 and U87MG tumor cells. To confirm the functional role of B18R as type I IFN inhibitor we tested two concentrations of B18R, i.e. 2 and 4 µg/mL to complex with NDV during infection in A549 cells. A549 cell line was chosen in this experiment as it showed good response to treatment with B18R (Fig. 2a). The result confirmed that B18R protein inhibited apoptosis in a dose-dependent manner in A549 cell lines (Fig. 2d).

### NDV-induced soluble mediators in the medium induces apoptosis and it is inhibited by B18R

**Figure 3:**
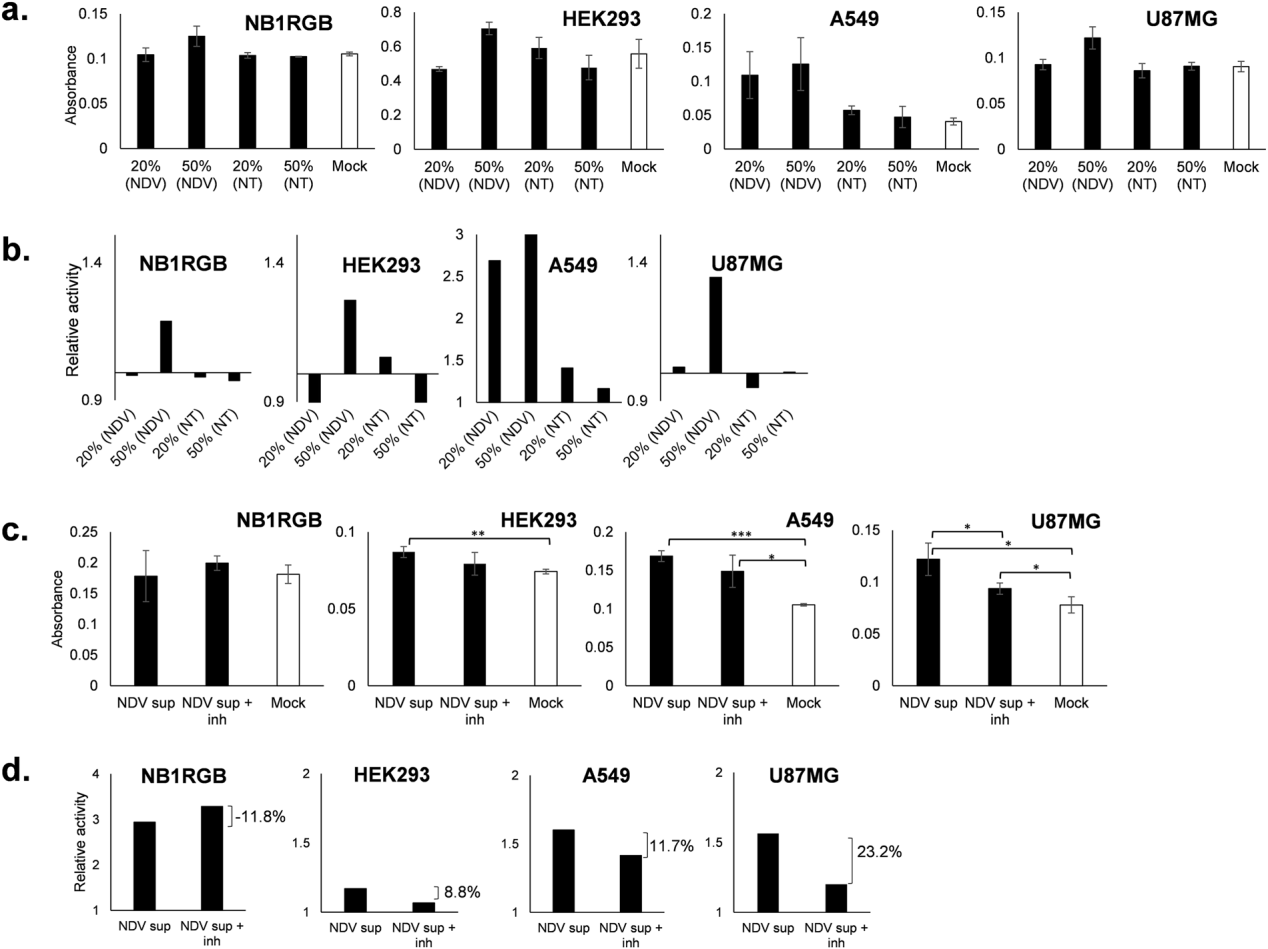
Apoptosis in normal and tumor cells induced by UV-inactivated NDV supernatant. Caspase-3 activity of normal NB1RGB and HEK293 cells, and tumor A549 and U87MG cells after treated with 20% or 50% v/v UV-inactivated culture medium from NDV-infected cells (**a**). A set of UV-inactivated supernatant from mock infected wells were also prepared. (**b**) Is the relative activity of treated to mock groups from data (**a**). Normal and tumor cells were treated with 100% v/v UV-inactivated supernatant and supplemented with B18R (**c**). The relative activity is represented in (**d**) and percentage of inhibition is noted. Error bar is standard deviation of absorbance from triplicate measurements. *P ≤ 0.05, **P < 0.01, ***P < 0.001.

To demonstrate that oncolysis was induced by soluble mediators and not a direct killing by virus replication, we prepared bioassay of supernatants from NDV-infected cells. First, NB1RGB, HEK293, A549 and U87MG cells were infected at 0.001 MOI NDV. Supernatants were collected at 48 hours, and any virus present was inactivated under UV light. Inactivation was confirmed by TCID_50_ after first passage in A549 cells. Inactivated supernatants were then applied to corresponding cell culture at 20% or 50% v/v (20% (NDV) and 50% (NDV), respectively). We also included 20% or 50% v/v inactivated supernatant from non-treated cells (20% (NT) and 50% (NT), respectively) to ensure that the observed effect was induced by NDV. Result shown in Fig. 3a,b indicated that all cell lines demonstrated apoptosis activity at concentration 50% v/v NDV supernatant. A549 cells was the most responsive cell type relative to the non-treated cells where high apoptosis level was detected even at 20% supernatant concentration. Most cell lines did not exhibit apoptosis activity at appreciable level when treated with supernatant from non-treated cells. Our results confirmed that apoptosis was driven by soluble mediators induced by NDV in a dose-dependent manner.

To confirm that oncolytic activity was secondary to IFNs induction, we included B18R protein into the bioassay. In this experiment we cultured cells in whole UV-inactivated supernatant (Fig. 3c,d) with and without B18R. Apoptosis activity was inhibited in tumor A549 and U87MG cells (11.7% and 23.2% inhibition, respectively), although significant difference between supernatant only and supernatant with inhibitor groups were observed only in U87MG tumor cells. Nevertheless, we ascertained that inhibition would be more significant when higher inhibitor concentration was added. Apoptosis inhibition was not observed in NB1RGB normal cells, suggesting that apoptosis in this cell line was independent from type I IFN signaling. Apoptosis inhibition was also observed in HEK293 cells (8.8% inhibition), but the relative activities were lower than those of tumor cells.

### Different action of type I and III IFNs for inducing apoptosis in normal and tumor cells

**Figure 4:**
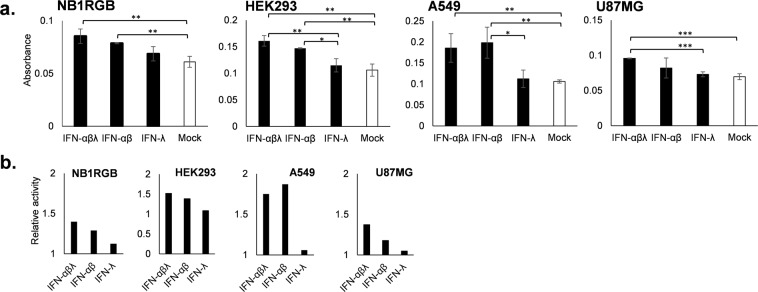
Apoptosis activity after different IFNs treatment. Normal cells (NB1RGB and HEK293) and tumor cells (A549 and U87MG) (**a**) were coincubated with combination of IFN type I and III (IFN-αβλ) or IFN type I only (IFN-αβ) or IFN type III only (IFN-λ) for 24 hours. Cells lysates were collected and tested for caspase-3 activity. The relative activity is the ratio of absorbance between treated and mock-treated cell. (**b**) Error bar is standard deviation of absorbance from triplicate wells. *P ≤ 0.05, **P < 0.01, ***P < 0.001.

To demonstrate the effect of type I and type III IFNs for inducing apoptosis, we incubated NB1RGB, HEK293, A549 and U87MG cells with combinations of IFN-α, -β and -λ (IFN-αβλ) or IFN-α and -β (IFN-αβ) or only IFN-λ for 24 hours. In all cell lines tested (Fig. 4a,b), highest apoptosis activity was observed in IFN-αβλ group and followed with IFN-αβ groups and the differences when compared to mock-treated cells were statistically significant. Meanwhile, treatment with IFN-λ showed very low caspase activity, indicating that IFN-λ alone, at such condition, has very low effect to induce apoptosis.

## Discussion

A paucity of information is available to explain the mechanisms of NDV tumor selective oncolysis. Impaired antiviral signaling in tumor cells, in particular interferons, has been proposed as the basis of NDV tumor selectivity^26–28,42^. However, many tumor cells with competent antiviral IFN signaling can still be killed by NDV^30,32,43^. Considering the potency of NDV to be as a strong type I and III IFN inducers, with much stronger induction of type I IFN was observed in tumor cells^25^; in this study we investigate the effect of NDV-induced antiviral interferons to stimulate apoptosis in tumor cells.

We demonstrated in the present work that oncolytic activity of NDV LaSota was a consequence of a selective induction of apoptosis in tumor cells despite the low virulence or low dose usage of the virus. We were able to confirm with TCID_50_ and flow cytometry of NDV antigen that our virus was lentogenic and the dose used was very low that was insufficient for the virus to replicate. In the next part, we infected normal (NB1RGB and HEK293) and tumor cells (A549 and U87MG) with NDV to observe apoptosis induction through the measurement of caspase-3 enzyme activity. We used a low MOI to ensure that cell death was unlikely because of virus replication. Result showed that NDV infection induced apoptosis in tumor A549 and U87MG cells unlike in normal cell HEK293. However, apoptosis was inhibited by B18R supplementation in A549 and U87MG tumor cells rather than in HEK293 normal cells. This suggests apoptosis induced by NDV infection was a type I IFN dependent and it is unique to tumor cells A549 and U87MG. B18R protein is a protein encoded by vaccinia virus that act as soluble IFN-α/β binding protein. The protein can bind to cell surface and inhibits the antiviral response signaled by IFN-α/β^40,41^. In this study we treated cells with B18R together with NDV during infection to ensure B18R binds to cell surface thus prevent IFN-α/β from initiating signal transduction leading to apoptosis. In addition, we observed a higher caspase activity in NB1RGB normal cells which is possibly a physiological apoptosis as a result of early NDV infection. Several studies have concluded that the fibroblast apoptosis is as a phase of tissue repair or wound healing after inflammation potentially because of the induction of cytokines, growth factors or extracellular matrices^44–46^. In our previous study, we have observed that NB1RGB exhibited relatively high cell death at 24 hours after NDV infection, but the cells viability would increase until 72 hours at when the observation was terminated^25^. In this study we observed that apoptosis was not inhibited by B18R supplementation in NB1RGB, confirming the tumor-specific type I IFN dependent apoptosis.

We further evaluated to determine if NDV-induced soluble mediators might account for apoptosis. Set of cell groups, normal and tumor, were treated with inactivated supernatants derived from NDV infected cells and apoptosis activity was measured. Apoptosis was observed in all cell types to a certain degree, however, they demonstrated apoptosis in dose-dependent fashion. This suggests the presence of apoptosis-inducing mediators contained in the media. Similar to former study with NDV infection, the addition of B18R into the medium was able to lower caspase-3 activity in A549 and U87MG tumor cells and not in NB1RGB normal cells. To simulate the content of interferons in the media, we also performed experiments on tumor and normal cells treated with commercially available recombinant IFN-α, -β and -λ. NB1RGB, HEK293, A549 and U87MG cells received combinations of interferons at the same concentration i.e. a total 3000 IU per well. Caspase activity assay showed that all cells treated with mixed IFN-α, -β and -λ exhibit higher caspase activity, followed by treatment with IFN-α, -β. Meanwhile, treatment with IFN-λ alone did not show any remarkable apoptosis activity in those cell lines.

Cells respond to virus infection by secreting mainly type I and III IFN antiviral cytokines. Although both types of IFNs act through distinct receptors, they induce similar signal transduction events, ISGs and biological activities *in vitro*^18,23^. Considering that NDV infection induces different IFNs secretion in tumor and normal cells, with the majority type I and III in tumor cell, and type III in normal cells^25^, here we provide evidence of different IFNs secretion regulates tumor selective apoptosis. Normal cells used in this study, i.e. NB1RGB and HEK293 secreted mainly IFN-λ with low or no IFN-α and -β, therefore they showed relatively weak apoptosis inhibition when B18R was added. In contrast, tumor cells such as A549 and U87MG that secreted high level of IFN-α, -β and -λ demonstrated significant apoptosis inhibition by B18R. Remarkably, experiments with commercially available IFNs confirmed that apoptosis was higher when IFN-α and -β were present, regardless of IFN-λ. Our results strongly suggest that NDV-induced apoptosis in current study is a type I IFN dependent process.

IFN-α induces apoptosis in malignant cells through the activation of caspases-1, 2, 3, 8 and 9 and independent of Fas signaling^39^. Furthermore, IFN-α and -β induce apoptosis through the upregulation of transcriptional activity of p53 tumor suppressor^21,22^. It was reported that IFN-λ also has the potential to induce apoptosis in colorectal carcinoma cell line^19^, however, in current study we observed only a little effect of IFN-λ to induce apoptosis in NB1RGB, HEK293, A549 and U87MG. Because in some studies, induction of apoptosis by interferons required more than 36 hours stimulation^19,47^, thus a longer incubation might be needed to see similar effect in other cell lines.

**Figure 5:**
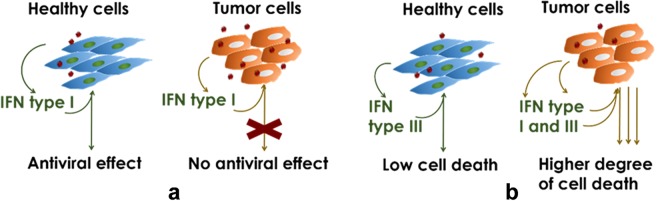
Model of NDV tumor-selectivity. (**a**) Previously proposed NDV tumor-selectivity was through the defective of type I IFN signaling in tumor cells^28^. (**b**) Higher death in tumor cells is due to cumulative effect of type I and III IFNs induced by NDV in tumor cells rather than normal cells.

In summary here, our data explain that NDV preferentially induces apoptosis in tumor cells because of the cumulative effects of IFNs, in particular IFN-α and -β, rather than impairment of IFN signaling (Fig. 5). In an *in vivo* condition, secreted IFN-α and -β by tumor cells might act in paracrine fashion to the surrounding tumor cells acting as tumor-selective apoptosis agent, thus we propose that NDV infection at a low MOI is a potent antitumor immunostimulation agent for cancer therapy.

Our study however does also present several limitations. First, we may need to test apoptosis activity in several time points and in escalating MOI to observe a much higher response. Although we were confident to see apoptosis effect at 24 hours after IFNs treatment as what was seen in NB1RGB and A549 cells, apparently it was not adequate for HEK293 and U878MG cells. Second, in our study we could not provide more elaborate flow cytometry data of cytotoxicity together with apoptosis. However, we have tested the cytotoxicity in our previous study^25^ and we are confident that NDV is tumor selective to all cell lines used in this study. Our findings suggest the importance of interferons’ role as antitumor in NDV infection which may not limited in apoptosis inducer only.

## Materials and Methods

### Cells

Human non-malignant cell lines i.e. NB1RGB (skin fibroblast) and HEK293 (transformed embryonic kidney) and tumor cell lines i.e. A549 (non-small lung carcinoma) and U87MG (glioblastoma multiforme) were purchased from RIKEN BioResource Center – Cell Bank (Tsukuba, Ibaraki, Japan) or American Type Culture Collection (ATCC, Rockville, MD, USA). A549 and U87MG tumor cell lines express high IFN-α, -β and -λ, while NB1RGB, HEK293 non-malignant cell lines express low IFN-α, -β and high IFN-λ after NDV infection, thus suitable for the study of IFN signaling^25^. A549 cells were grown in F12-K medium; U87MG and HEK293 cells in DMEM high glucose medium; NB1RGB cells in MEMα medium. All growth media were supplemented with 10% fetal bovine serum (FBS), 100 IU/mL penicillin and 100 µg/mL streptomycin. Media and antibiotics were obtained from Gibco (Carlsbad, CA, USA) and FBS was obtained from (Biowest, Nuaillé, France). Cells were cultured at 37 °C in 5% CO_2_.

### Virus

Lentogenic NDV LaSota strain was received from PT. IPB-Shigeta Animal Pharmaceuticals, Bogor, Indonesia, which is used for anti-NDV vaccine production. Virus was inoculated in the allantoic cavity of 10-day-old embryonated chicken eggs. Eggs were incubated at 37 °C for 48 hours, followed by embryo inactivation at −4 °C overnight. Possibly contaminated eggs with appearance such as cloudy or reddish allantoic fluid or broken yolk, were discarded. Allantoic fluid was collected and cleared from debris at 5000 × *g*, 4 °C, for 30 minutes. Titer of infectious particle was confirmed by end point dilution assay tissue culture infective dose 50 (TCID_50_) according to previous method with modification^25^. Collected allantoic fluid was tested for microbial contaminants by inoculation in TSA (tryptone soy agar) containing lecithin and polysorbate prepared according to manufacturer’s protocol (Microbial Content Test Agar – Scharlau, Barcelona, Spain) (Supplementary Informations, Fig. S3). All experiment using living virus and cell culture was conducted in biosafety level-2 containment of Mochtar Riady Institute for Nanotechnology (MRIN) and approved by MRIN ethics committee (Protocol number: 01.1312012).

### NDV infectivity in tumor and normal cells

Confluent normal and tumor cells were set up in a 6-well plate. Plates were washed with PBS, then infected with NDV at multiplicity of infection (MOI) 0.001 for 60 minutes at 37 °C. Excess virus was removed and cells were washed. Cultures were then overlaid with maintenance medium (similar to growth medium except that FBS was replaced with 0.3% BSA Fraction V (Gibco). A hundred microliter culture supernatants were collected at 24, 48, and 72 hours post-infection and stored at −80 °C upon use for virus growth assay and apoptosis assay. For virus growth assay_,_ supernatants from each time point were ten-fold serially diluted and titred for virus in A549 cells with end point dilution assay. After 5 days incubation, plates were formalin-inactivated and incubated with mouse monoclonal anti-NDV immunoglobulin (IgG) (Abcam, Cambridge, UK) 1:200 dilution, followed by incubation with goat anti-mouse IgG alexa fluor 488-conjugated (Invitrogen, Carlsbad, CA, USA). Fluorescence was measured in Varioskan Lux microplate reader (Thermo Fisher Scientific, Singapore). TCID_50_ value was calculated with Reed and Muench method^48^.

### Flow cytometry analysis of NDV proteins on infected cells

Normal and tumor cells were infected with NDV at 0.001 MOI for 5 minutes or 24 hours. For 5 minutes infection, cells were washed after 5 minutes with PBS twice and then detached with Accutase (Gibco). For 24 hours infection, cells were incubated with virus for 60 minutes at 37 °C, washed with PBS twice, resuspended with maintenance medium and incubated for 24 hours at 37 °C. Untreated cells were prepared for infection negative control. Medium was removed and cells were detached with Accutase. Any infectious virus was inactivated with 10% formalin and then cells were washed. For the detection of NDV protein on cell surface, cells were incubated with mouse monoclonal anti-NDV (6H12) alexa fluor 488-conjugated (Novus Biologicals, LLC, Littleton, CO, USA) at 1:50 dilution for 30 minutes at room temperature. The level of surface NDV antigen was analyzed in FITC green stain FL1 channel of flow cytometer BD Accuri (Becton Dickinson, Franklin Lakes, NJ, USA). Data were analysed using CSampler software (Becton Dickinson).

### Immunofluorescence assay

A total of 1 × 10 of normal and tumor cells were seeded in a 2 × 2 cm sterile coverslips a day before treatment. On the next day, cells were infected with 0.001 MOI NDV diluted with maintenance medium for 60 minutes at 37 °C. Cells were then washed, media were replaced with maintenance medium and cover slips were incubated for 24 hours at 37 °C. Cover slips were washed 3 times with PBS and fixed in 10% formalin for 30 minutes, followed with PBS wash and incubation with mouse monoclonal anti-NDV (6H12) alexa fluor 488-conjugated (Novus Biologicals) in PBS (1:1000) for 60 minutes in dark at room temperature. Cover slips were washed with PBS and incubated with 4′6-diamidino-2-phenylindole (DAPI) (Cell Signaling Technology, Danvers, MA, USA) diluted in PBS to bring to 1 µg/mL final concentration at room temperature for 5 minutes, followed with PBS wash and observation under fluorescent microscope. NDV antigen was observed in FITC channel, while nucleus counterstaining was observed in DAPI channel (Supplementary Informations, Fig. S1).

### Caspase-3 enzyme activity assay in NDV-infected cells

Activity of apoptosis was evaluated by measuring activation of caspase-3 enzyme using CaspACE assay System (Promega, Madison, WI, USA). Cells (5 × 10^5^) in each well of a 12-well plate were infected with NDV at 0.001 MOI treated with B18R recombinant protein (eBioscience – Thermo Fisher Scientific, Singapore), a type I IFN inhibitor. Virus suspension was pre-incubated with B18R for 30 minutes to bring to room temperature, followed with 60 minutes infection at 37 °C. Cells were then incubated in maintenance medium for 24 hours (or 36 hours for U87MG cells) at 37 °C. U87MG cells required longer incubation for observable color change. Mock-treated cells were prepared for negative control. Cells were harvested with Accutase, washed and proteins were extracted in 70 µL of cell lysis buffer provided in the kit. Protein concentration of lysate was measured by using Quick Start Bradford Dye reagent (Bio-Rad, Hercules, CA, USA). Caspase-3 activity was measured by mixing 50–100 µg protein extracts with caspase assay buffer (Promega), dimethylsulphoxide/DMSO (VWR International, Solon, OH, USA), dithiothreitol/DTT (Bio-Rad) 100 mM and AC-DEVD-pNA substrate (Promega) 0.2 mM in total 100 µL reaction mixture. Mixture was incubated at room temperature overnight and free pNA level was measured at 405 nm. Each assay was conducted using protein extracts at the same concentration. Data were taken from triplicate reaction.

### NDV supernatant assay

Cells were washed and then infected with NDV at 0.001 MOI. Mock-infected wells were treated with PBS. Culture supernatant was collected at 48 hours p.i. and UV-exposed for 30 minutes at room temperature for inactivation of virus. To confirm that virus was inactivated well, supernatant was passaged in A549 cells once and supernatant was evaluated by TCID_50_^49^. Supernatant was added to newly prepared cell culture in a 12-well plate at a concentration of 20% or 50% v/v (supernatant:fresh maintenance medium). Final volume of medium was 1 mL. For experiment using B18R, an undiluted (100% v/v) UV-inactivated supernatant was used. Plates were incubated for 24 hours at 37 °C and cell lysate was subjected to caspase-3 colorimetric activity (Promega).

### Interferons combination assay

Human interferons used were commercially available recombinant proteins i.e. IFN-α (PBL Assay Science, Piscataway, NJ, USA), IFN-β (R&D System, MN, USA) and IFN-λ (PBL). Confluent normal and tumor cell lines were prepared in a 12-well plates. Cells were washed and medium was replaced with fresh maintenance medium supplemented with IFN-α, -β and -γ (1000 IU each) or IFN-α, -β (1500 IU each) or IFN-γ (3000 IU) in 1 mL medium. Plates were then incubated for 24 hours at 37 °C and cell lysate was subjected to caspase-3 activity (Promega).

### Annexin-V flow cytometry

For detection of apoptosis, cells were detached with Accutase (Gibco) and washed twice in PBS. Cells were incubated at room temperature with Annexin V-FITC (Cell signaling, Danvers, MA, USA) in 1:25 dilution in binding buffer for 30 minutes. Cells were then treated with 10% formalin for another 30 minutes to inactivate virus^49^. Cells were washed, resuspended with PBS and then analyzed in BD Accuri flow cytometer (Becton Dickinson). Data were processed in CSampler software (Becton Dickinson).

### Statistics

Data were analyzed by two-tailed unpaired Student’s t test for groups comparing NDV to mock group. The P-values of ≤0.05 was considered statistically significant (*P ≤ 0.05, **P < 0.01, ***P < 0.001, ****P < 0.0001).

## Supplementary information

Antiviral interferons induced by Newcastle disease virus (NDV) drive a tumor-selective apoptosis

## References

[1] Laurie SA (2006). L. R. A Phase 1 Clinical Study of Intravenous Administration of PV701, an Oncolytic Virus, Using Two-Step Desensitization. Clin. Cancer Res..

[2] Freeman A (2006). Phase I/II Trial of Intravenous NDV-HUJ Oncolytic Virus in Recurrent Glioblastoma Multiforme. Mol. Ther..

[3] Zamarin D (2014). Localized oncolytic virotherapy overcomes systemic tumor resistance to immune checkpoint blockade immunotherapy. Sci. Transl. Med..

[4] Pecora AL (2002). L. R. Phase I Trial of intravenous administration of PV701, an oncolytic virus, in patients with advanced solid cancers. J. Clin. Oncol..

[5] Yaacov, B., Elihaoo, E., Greenbaum, I. & Panet, A. Selective oncolytic effect of an attenuated Newcastle disease virus (NDV-HUJ) in lung tumors. 795–807, 10.1038/cgt.2008.31 (2008). 10.1038/cgt.2008.31 18535620

[6] Phuangsab A, Lorence RM, Reichard KW, Peeples ME, Walter RJ (2001). Newcastle disease virus therapy of human tumor xenografts: antitumor effects of local or systemic administration. Cancer Lett..

[7] Song KY (2010). Antitumor efficacy of viral therapy using genetically engineered Newcastle disease virus [NDV(F3aa)-GFP] for peritoneally disseminated gastric cancer. J. Mol. Med..

[8] Lorence RM (1994). Complete regression of human fibrosarcoma xenografts after local Newcastle disease virus therapy. Cancer Res..

[9] Lorence RM (1994). Complete regression of human neuroblastoma xenografts in athymic mice after local Newcastle disease virus therapy. J. Natl. Cancer Inst..

[10] Farkona S, Diamandis EP, Blasutig IM (2016). Cancer immunotherapy: The beginning of the end of cancer?. BMC Med..

[11] Isaacs A, Lindenmann J (1957). Virus Interference. I. The Interferon. Proc. R. Soc. B Biol. Sci..

[12] Levy DE, Marié IJ, Durbin JE (2011). Induction and Function of Type I and III Interferon in Response to Viral. Infection. Curr. Opin. Virol..

[13] Ank N (2006). Lambda interferon (IFN-λ), a type III IFN, is induced by viruses and IFNs and displays potent antiviral activity against select virus infections *in vivo*. J. Virol..

[14] Jarahian M (2009). Activation of natural killer cells by newcastle disease virus hemagglutinin-neuraminidase. J. Virol..

[15] Hemann, E. A., Gale, M. & Savan, R. Interferon lambda genetics and biology in regulation of viral control. *Front. Immunol*. **8** (2017). 10.3389/fimmu.2017.01707 PMC5723907 29270173

[16] Batliwalla FM (1998). A 15-year follow-up of AJCC stage III malignant melanoma patients treated postsurgically with Newcastle disease virus (NDV) oncolysate and determination of alterations in the CD8 T cell repertoire. Mol. Med..

[17] Smyth MJ (2005). Type I interferon and cancer immunoediting. Nat. Immunol..

[18] Garcia-Diaz A (2017). Interferon Receptor Signaling Pathways Regulating PD-L1 and PD-L2 Expression. Cell Rep..

[19] Li W, Lewis-Antes A, Huang J, Balan M, Kotenko SV (2008). Regulation of apoptosis by type III interferons. Cell Prolif..

[20] Parker BS, Rautela J, Hertzog PJ (2016). Antitumour actions of interferons: Implications for cancer therapy. Nat. Rev. Cancer.

[21] Takaoka A (2003). Integration of interferon-α/β signalling to p53 responses in tumour suppression and antiviral defence. Nature.

[22] Porta C (2005). Interferons α and γ induce p53-dependent and p53-independent apoptosis, respectively. Oncogene.

[23] Muñoz-Fontela C (2008). Transcriptional role of p53 in interferon-mediated antiviral immunity. J. Exp. Med..

[24] Zhou Z (2007). Type III interferon (IFN) induces a type I IFN-like response in a restricted subset of cells through signaling pathways involving both the Jak-STAT pathway and the mitogen-activated protein kinases. J. Virol..

[25] Ginting T, Suryatenggara J, Christian S, Mathew G (2017). Proinflammatory response induced by Newcastle disease virus in tumor and normal cells. Oncolytic Virotherapy.

[26] Wagner TC (2004). Interferon receptor expression regulates the antiproliferative effects of interferons on cancer cells and solid tumors. Int. J. Cancer.

[27] Katsoulidis E, Kaur S, Platanias LC (2010). Deregulation of interferon signaling in malignant cells. Pharmaceuticals.

[28] Fiola C (2006). Tumor selective replication of Newcastle disease virus: association with defects of tumor cells in antiviral defence. Int J Cancer.

[29] Krishnamurthy S, Takimoto T, Scroggs RA, Portner A (2006). Differentially Regulated Interferon Response Determines the Outcome of Newcastle Disease Virus Infection in Normal and Tumor Cell Lines. J. Virol..

[30] Lazar I (2010). The Oncolytic Activity of Newcastle Disease Virus NDV-HUJ on Chemoresistant Primary Melanoma Cells Is Dependent on the Proapoptotic Activity of the Inhibitor of Apoptosis Protein Livin. J. Virol..

[31] Vigil A, Martinez O, Chua MA, García-Sastre A (2008). Recombinant Newcastle disease virus as a vaccine vector for cancer therapy. Mol. Ther..

[32] Vigil A (2007). Use of reverse genetics to enhance the oncolytic properties of newcastle disease virus. Cancer Res..

[33] Molouki A, Hsu YTe, Jahanshiri F, Rosli R, Yusoff K (2010). Newcastle disease virus infection promotes Bax redistribution to mitochondria and cell death in HeLa cells. Intervirology.

[34] Molouki A, Yusoff K (2012). NDV-induced apoptosis in absence of Bax; Evidence of involvement of apoptotic proteins upstream of mitochondria. Virol. J..

[35] Elankumaran S (2006). Newcastle Disease Virus Exerts Oncolysis by both Intrinsic and Extrinsic Caspase-Dependent Pathways of Cell Death Newcastle Disease Virus Exerts Oncolysis by both Intrinsic and Extrinsic Caspase-Dependent Pathways of Cell Death..

[36] Fábián Z, Csatary CM, Szeberényi J, Csatary LK (2007). p53-independent endoplasmic reticulum stress-mediated cytotoxicity of a Newcastle disease virus strain in tumor cell lines. J. Virol..

[37] Ghrici, M., Zowalaty, M. E. L., Omar, A. R. & Ideris, A. Induction of apoptosis in MCF-7 cells by the hemagglutinin-neuraminidase glycoprotein of Newcastle disease virus Malaysian strain AF2240. 1035–1044, 10.3892/or.2013.2573 (2013). 10.3892/or.2013.2573 PMC3783058 23807159

[38] Mansour M, Palese P, Zamarin D (2011). Oncolytic Specificity of Newcastle Disease Virus Is Mediated by Selectivity for Apoptosis-Resistant Cells. J. Virol..

[39] Thyrell L (2002). Mechanisms of interferon-alpha induced apoptosis in malignant cells. Oncogene.

[40] Alcamí A, Symons JA, Smith GL (2000). The vaccinia virus soluble alpha/beta interferon (IFN) receptor binds to the cell surface and protects cells from the antiviral effects of IFN. J. Virol..

[41] Colamonici OR, Domanski P, Sweitzer SM, Larner A, Buller RML (1995). Vaccinia virus B18R gene encodes a type I interferon-binding protein that blocks interferon α transmembrane signaling. Journal of Biological Chemistry.

[42] Reichard KW (1992). Newcastle disease virus selectively kills human tumor cells. J. Surg. Res..

[43] Puhlmann J, Puehler F, Mumberg D, Boukamp P, Beier R (2010). Rac1 is required for oncolytic NDV replication in human cancer cells and establishes a link between tumorigenesis and sensitivity to oncolytic virus. Oncogene.

[44] Jelaska A, Korn JH (1998). Anti-Fas induces apoptosis and proliferation in human dermal fibroblasts: Differences between foreskin and adult fibroblasts. J. Cell. Physiol..

[45] Santiago B, Galindo M, Palao G, Pablos JL (2004). Intracellular Regulation of Fas-Induced Apoptosis in Human Fibroblasts by Extracellular Factors and Cycloheximide. J. Immunol..

[46] Fluck J (1998). Normal human primary fibroblasts undergo apoptosis in three-dimensional contractile collagen gels. J. Invest. Dermatol..

[47] Thyrell L (2004). Interferon alpha-induced apoptosis in tumor cells is mediated through the phosphoinositide 3-kinase/mammalian target of rapamycin signaling pathway. J. Biol. Chem..

[48] Reed LJ, Muench H (1938). A simple method of estimating fifty percent endpoints. Am. J. Epidemiol..

[49] Lifson JD, Sasaki DT, Engelman EG (1986). Utility of formaldehyde fixation for flow cytometry and inactivation of the AIDS associated retrovirus. J. Immunol. Methods.

