# Supplementary material for: Antiviral interferons induced by Newcastle disease virus (NDV) drive a tumor-selective apoptosis
Source: Sci Rep. 2019 Oct 22;9:15160. doi: 10.1038/s41598-019-51465-6 (PMC6806003; doi:10.1038/s41598-019-51465-6)
Supplement: Supplementary file 1 — Antiviral interferons induced by Newcastle disease virus (NDV) drive a tumor-selective apoptosis [file 41598_2019_51465_MOESM1_ESM.pdf]

Supplementary informations:

Antiviral interferons induced by Newcastle disease virus (NDV) drive a tumor-selective apoptosis

Teridah Ernala Ginting[1],* Salomo Christian[1], Young Othiwi Larasati[1], Jeremiah Suryatenggara[1], Ivet Suriapranata[1], George Mathew[1]

[1]Division of Immunology, Mochtar Riady Institute for Nanotechnology and Medical Science Group, University of Pelita Harapan. Jalan Boulevard Jenderal Sudirman 1688, Lippo Karawaci, Tangerang, Banten 15811. Phone: (62) 21-54210123.

*Corresponding author: E-mail: tginting@mrinstitute.org

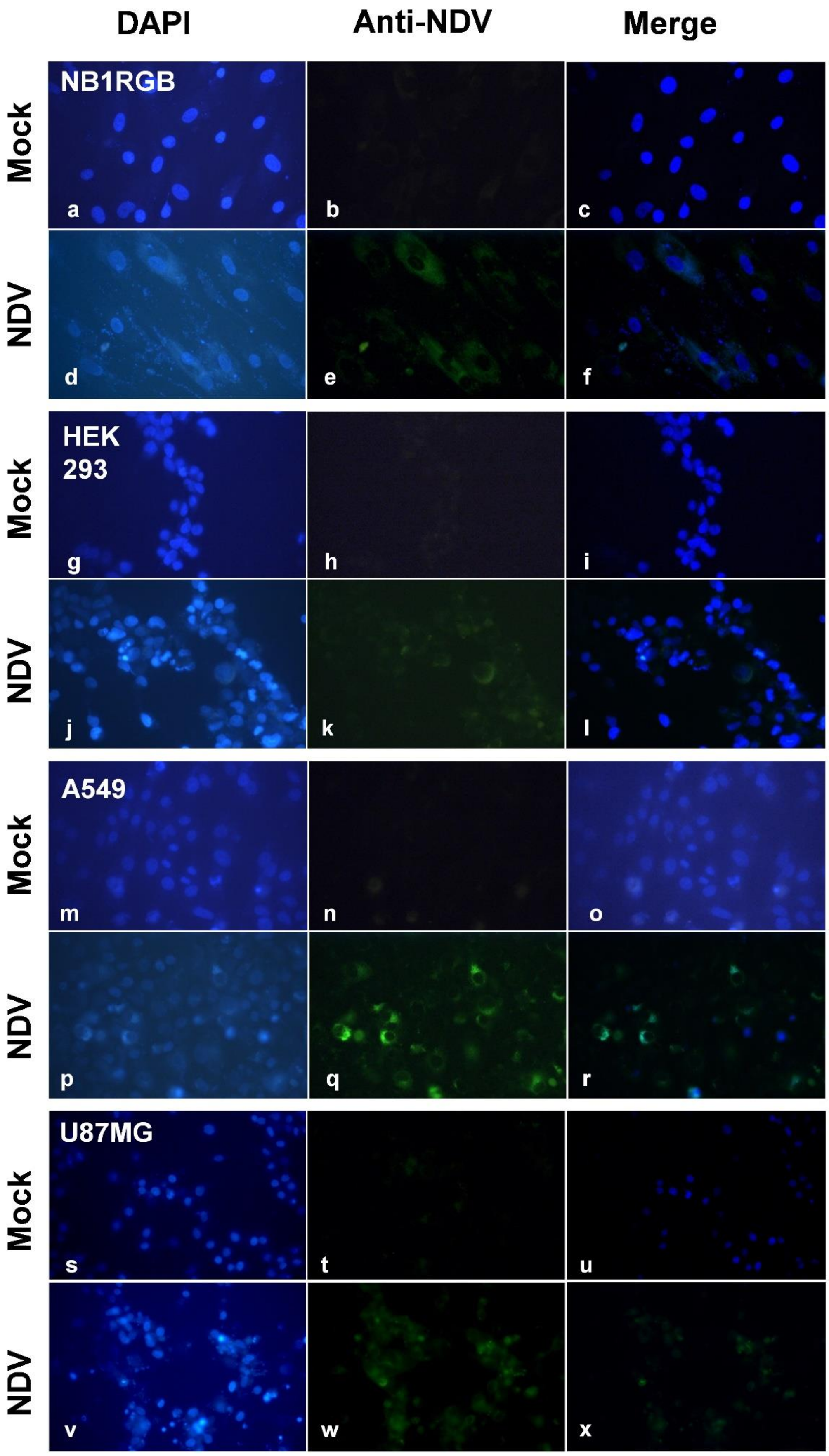
DAPI
Anti-NDV
Merge
Mock
NDV
Mock
NDV
Mock
NDV
Mock
NDV
NB1RGB
HEK
293
A549
U87MG
a
b
c
d
e
f
g
h
i
j
k
l
m
n
o
p
q
r
s
t
u
v
w
x

Figure S1 Immunofluorescence staining of NDV protein on normal and tumor cells 24 hours pi. Normal (NB1RGB and HEK293) cell lines and tumor (A549 and U87MG) cell lines were infected with NDV at 0.001 MOI and NDV protein was detected with alexa fluor-488-conjugated NDV antibody (anti-NDV) after 24 hours. Mock infected cells were provided as negative control. DAPI was used for counterstaining. Merge is the overlay of DAPI and anti-NDV images taken from the same field of view. NDV protein expressed on the membrane of NDV-infected cells was shown as green color.

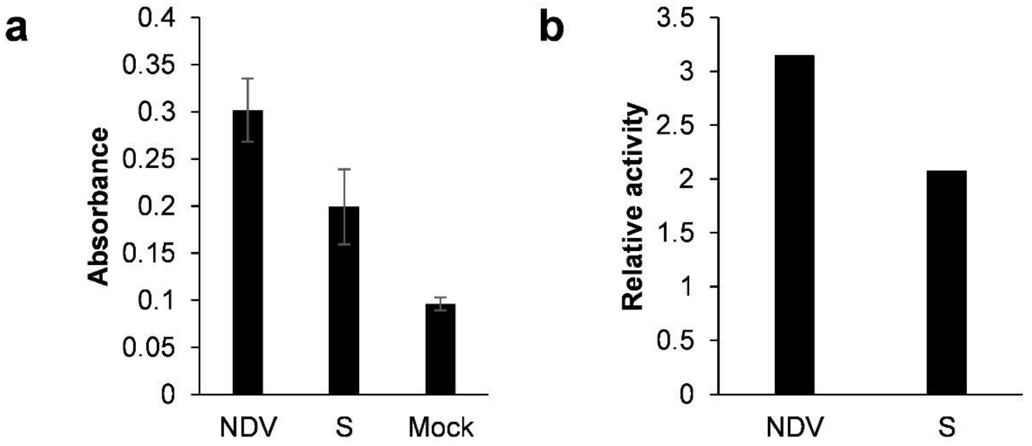

Figure S2 NDV at 0.001 MOI is enough to induce apoptosis. NDV infection at 0.001 MOI (NDV) induced apoptosis in A549 cells as measured by the activation of caspase-3 enzyme. Positive control apoptosis was cells treated with 1 μM of staurosporine for 24 hours (S). Figure (a) is absorbance value and (b) is relative activity or ratio of treated cells to mock.

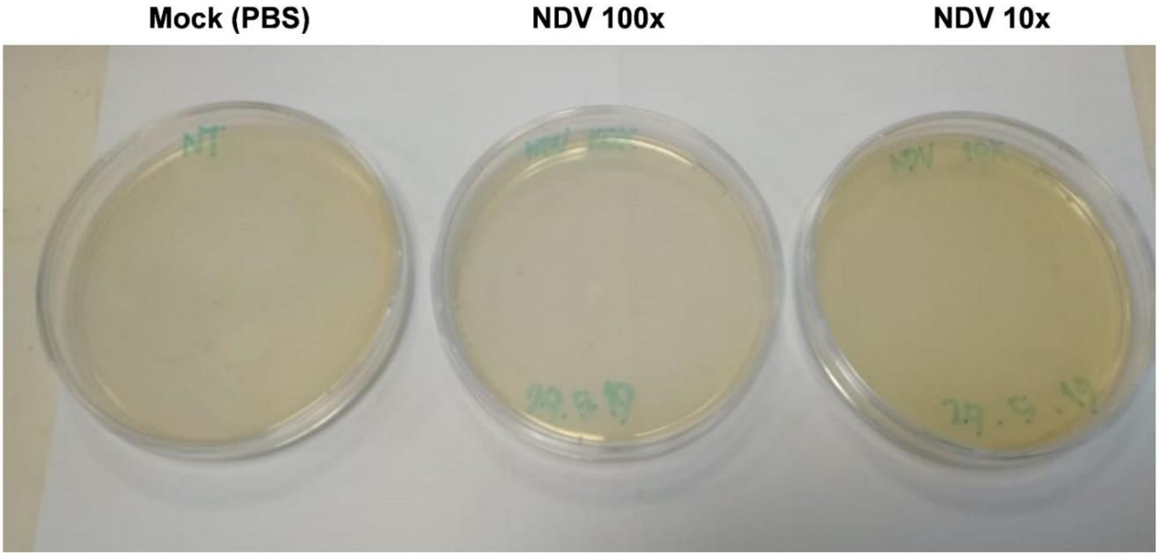

Figure S3 Contaminant test on prepared allantoic fluid showed negative contamination. Tryptone soy agar (TSA) containing tryptone, soy, lecithin and polysorbate were inoculated with 10 μL allantoic fluid collected from NDV inoculated eggs, at 10 or 100 times dilution in PBS (NDV 10x and NDV 100x, respectively). Control plate was inoculated with 10 μL PBS (Mock). Agar plates were then incubated for 48 hours at 37°C.
